# The effects of 2,3,7,8-tetrachlorodibenzo-*p*-dioxin (TCDD) on the transcriptome of aryl hydrocarbon receptor (AhR) knock-down porcine granulosa cells

**DOI:** 10.7717/peerj.8371

**Published:** 2020-01-22

**Authors:** Monika Ruszkowska, Agnieszka Sadowska, Anna Nynca, Karina Orlowska, Sylwia Swigonska, Tomasz Molcan, Lukasz Paukszto, Jan P. Jastrzebski, Renata E. Ciereszko

**Affiliations:** 1 Department of Animal Anatomy and Physiology, Faculty of Biology and Biotechnology, University of Warmia and Mazury in Olsztyn, Olsztyn, Poland; 2 Laboratory of Molecular Diagnostics, Faculty of Biology and Biotechnology, University of Warmia and Mazury in Olsztyn, Olsztyn, Poland; 3 Department of Plant Physiology, Genetics and Biotechnology, Faculty of Biology and Biotechnology, University of Warmia and Mazury in Olsztyn, Olsztyn, Poland

**Keywords:** NGS, Porcine granulosa cells, TCDD, Transcriptome, AhR knock-down

## Abstract

**Background:**

2,3,7,8-tetrachlorodibenzo-*p*-dioxin (TCDD) is a toxic man-made chemical, adversely affecting reproductive processes. The well-characterized canonical mechanism of TCDD action involves the activation of aryl hydrocarbon receptor (AhR) pathway, but AhR-independent mechanisms were also suggested. By applying RNA interference technology and Next Generation Sequencing (NGS) we aimed to identify genes involved in the mechanism of TCDD action in AhR knock-down porcine granulosa cells.

**Methods:**

Porcine granulosa cells were transfected with small interfering RNAs targeting mRNA of AhR. After transfection, medium was exchanged and the AhR knock-down cells were treated with TCDD (100 nM) for 3, 12 or 24 h, total cellular RNA was isolated and designated for NGS. Following sequencing, differentially expressed genes (DEGs) were identified. To analyze functions and establish possible interactions of DEGs, the Gene Ontology (GO) database and the Search Tool for the Retrieval of Interacting Genes (STRING) database were used, respectively.

**Results:**

The AhR gene expression level and protein abundance were significantly decreased after AhR-targeted siRNAs transfection of the cells. In TCDD-treated AhR knock-down cells we identified 360 differentially expressed genes (DEGs; *P*-adjusted < 0.05 and log2 fold change [log2FC] ≥ 1.0). The functional enrichment analysis of DEGs revealed that TCDD influenced the expression of genes involved, among other, in the metabolism of vitamin A, follicular development and oocyte maturation, proliferation and differentiation as well as inflammation, stress response, apoptosis and oncogenesis. The three-time point study demonstrated that TCDD-induced changes in the transcriptome of AhR knock-down porcine granulosa cells were especially pronounced during the early stages of the treatment (3 h).

**Conclusions:**

TCDD affected the transcriptome of AhR knock-down porcine granulosa cells. The molecules involved in the AhR-independent action of TCDD were indicated in the study. The obtained data contribute to better understanding of molecular processes induced by xenobiotics in the ovary.

## Introduction

Polychlorinated dibenzo-*p*-dioxins (PCDDs, dioxins) are highly toxic pollutants which arise from the combustion of chlorine-containing compounds and are widely distributed in the environment. They are by-products of various industrial processes and waste incineration. The most toxic PCDD congener is 2,3,7,8-tetrachlorodibenzo-*p*-dioxin (TCDD). Due to its high lipophilicity and resistance to biodegradation, TCDD accumulates in animal tissues (Larsen, 2006; Kulkarni, Crespo & Afonso, 2008). The half-life of TCDD in humans is estimated to be 7–11 years (Milbrath et al., 2009).

The canonical mechanism of TCDD action is associated with the activation of aryl hydrocarbon receptor (AhR) pathway. Following TCDD binding, AhR translocates to the nucleus where it dimerizes with aryl hydrocarbon receptor nuclear translocator (ARNT). The TCDD/AhR/ARNT heterodimer binds to dioxin response elements located in the regulatory region of target genes including those encoding enzymes involved in dioxin biodegradation (e.g., cytochrome P450, family 1, member A1 [*CYP1A1*], A2 [*CYP1A2* ] or B1 [*CYP1B1* ]). The increase in CYP1A1 expression is a molecular marker of TCDD action. The TCDD activation of AhR signaling pathway has been intensively studied in various cells and tissues of different species, including pigs (reviewed by Pohjanvirta, 2012). Other signaling pathways activated by TCDD, i.e., signaling pathways that are not mediated by AhR, were reported but they require more supportive data (Swedenborg & Pongratz, 2010; Matsumura, 2011; Wang et al., 2012; Ghotbaddini & Powell, 2015; Larigot et al., 2018).

The toxic effects of TCDD exerted on living organisms include immunotoxicity, hepatotoxicity and neurotoxicity. The dioxin was also found to cause reproductive defects (endometriosis, teratogenesis, abortion, diminished fertility) and endocrine disruption affecting e.g., luteal and follicular steroidogenesis (Petroff et al., 2001; Gregoraszczuk, 2002; Morán et al., 2003; Franczak et al., 2006). Ovarian granulosa cells which constitute, together with theca cells, the wall of the ovarian follicle play a crucial role in maintaining female fertility. They nurture oocytes and produce steroid hormones which ensure optimal conditions for reproductive performance (Albertini et al., 2001). Disruption of steroid hormone synthesis, activity or metabolism may lead to follicular dysfunction and atresia, affecting all reproductive processes in females (Sanderson, 2006).

Since TCDD influences the production of estrogens and progesterone by porcine granulosa cells (Gregoraszczuk, 2002; Jablonska et al., 2011; Jablonska et al., 2014), it is of importance to identify its molecular targets in follicular cells. The results of our previous studies, performed on porcine granulosa cells, demonstrated, among others, that TCDD affected the expression of transcripts involved in the follicular atresia as well as cell proliferation and cell cycle regulation (Sadowska et al., 2017; Ruszkowska et al., 2018; Nynca et al., 2019). The granulosa cells of pigs were found to express the Ah receptor (Sadowska et al., 2015). The aim of the present study was to answer the question whether TCDD may affect the transcriptional profile of porcine granulosa cells in an AhR-independent manner. Therefore we intended, for the first time, to examine the effects of TCDD action in AhR knock-down porcine granulosa cells. To meet this goal we applied RNA interference (RNAi) technology and Next Generation Sequencing (NGS).

## Materials & Methods

### Culture of porcine granulosa cells (AVG-16 cells)

AVG-16 cell line obtained from granulosa cells of pigs was purchased from The European Collection of Authenticated Cell Cultures (06062701; Salisbury, UK; Horisberger, 2006). Previously, we demonstrated that AVG-16 cells are an useful model for studying dioxin effects on ovarian functions (Sadowska et al., 2015). AVG-16 cells were cultured and passaged as previously described (Sadowska et al., 2015; Sadowska et al., 2017). Specifically, one day prior to siRNA transfection, cells were seeded in six-well culture plates with density of 0.7 × 10^6^ cells/three mL Dulbecco’s modified Eagle’s medium (Sigma Aldrich, St. Louis, MO, USA). At ∼70% confluency, the AVG-16 cells were washed (PBS) and the medium was exchanged. The cells were transfected with small interfering RNAs (siRNAs). Untransfected cells (C_UT_) were used as control.

### AhR gene knock-down in porcine granulosa cells

The granulosa cells were transfected as described previously (Orlowska et al., 2019). Specifically, for the transfection we used Viromer^®^ BLUE (Lipocalyx GmbH, Halle, Germany) and the mixture of three different siRNAs (anti-*AhR* 1 + anti-*AhR* 2 + anti-*AhR* 3; Sigma Aldrich; Table S1). Negative control cells (C_NEG_) were transfected with nontargeted siRNA (Invitrogen, Carlsbad, CA, USA). The transfection mixture was added to the cells in a drop-wise manner and then the cells were cultured for 24 h (37 °C, 5% CO_2_, 95% air). Untransfected cells (C_UT_) were used as controls. To check the efficacy of the AhR knock-down, the AhR gene expression level and protein abundance were determined in C_UT_ cells, C_NEG_ cells and cells transfected with the three relevant siRNA sequences (C_S_) by real-time PCR and western blotting, respectively.

### TCDD treatment of granulosa cells

In the current study, we compared the transcriptomes of the TCDD-treated AhR knock-down porcine granulosa cells and the untreated AhR knock-down cells. To better recognize the TCDD mode of action, the comparison was made at three time points: 3, 12 and 24 h of cell incubations with TCDD (Sadowska et al., 2017; Nynca et al., 2019). Due to the fact that two examined cell types (TCDD-treated and untreated cells) were both AhR knock-down, the effect of AhR knock-down was excluded. Such an approach was designed to capture only TCDD-induced changes in gene expression. After 24 h of cell transfection with the mixture of siRNAs (anti-*AhR* 1 + anti-*AhR* 2 + anti-*AhR* 3) fresh medium was added and the AhR knock-down cells were treated with 0 nM (control cells) or 100 nM of TCDD (TCDD-treated cells; Sigma Aldrich) for 3, 12 or 24 h (n = two biological replicates per one time point), yielding 12 examined samples. In the present study, we were focused on identifying all possible pathways (molecules) involved in the mechanism of TCDD action in porcine granulosa cells and, therefore, the selected dose of TCDD (100 nM) moderately exceeded its environmentally relevant concentrations. The concentration of 100 nM of TCDD was previously reported to affect porcine granulosa cells steroidogenesis (Gregoraszczuk et al., 2000; Gregoraszczuk, 2002; Jablonska et al., 2014), gene expression (Piasecka-Srader et al., 2016; Sadowska et al., 2017; Nynca et al., 2019), lncRNA expression (Ruszkowska et al., 2018) and proteome (Orlowska et al., 2018) without affecting cell viability and morphology. Moreover, 100 nM was one of the two most frequently used TCDD concentrations in *in vitro* experiments performed on porcine ovarian cells. After incubation, the medium was removed, cells were washed (PBS) and total RNA was isolated.

### Total RNA isolation and evaluation of RNA integrity

Total RNA isolation and evaluation of RNA integrity were performed as described previously (Sadowska et al., 2017; Ruszkowska et al., 2018). The RNA samples with integrity number greater than 8.0 were designated for NGS.

### Construction and sequencing of Illumina cDNA libraries

Paired-end cDNA libraries were prepared from purified RNA using Illumina TruSeq Stranded mRNA Sample Preparation Kit (Illumina Inc., San Diego, CA, USA) according to the manufacturer’s protocol. In brief, poly(T) magnetic beads were used to purify mRNA from total RNA sample. The first strand of cDNA was synthesized from the cleaved mRNA fragments using random-sequence primers and reverse transcriptase. In the next step, the RNA template was removed and the second strand of cDNA, with dUTP in place of dTTP, was synthesized by DNA polymerase I. Then, a single “A” nucleotide was added to the 3′ end of double-stranded cDNA, and cDNA was subjected to the adapter ligation. To create final libraries, cDNA samples were amplified through 15 cycles of PCR. The libraries’ profiles were validated using 2100 Bioanalyzer (Agilent Technologies, Santa Clara, CA, USA) to check the size distribution of the libraries. The Qubit High Sensitivity kit (Thermofisher Scientific, Waltham, MA, USA) was used to check the concentration of the samples. Finally, libraries were sequenced on HiSeq2000 instrument (Illumina) using 100 bp paired-end sequencing. The library construction and sequencing were performed by Source BioScience (Nottingham, UK).

### Bioinformatic analysis of gene expression

Following sequencing, the obtained cDNA fragments were evaluated and trimmed as previously described (Sadowska et al., 2017). The obtained fragments were mapped to the porcine reference genome with GTF annotation files (Sus Scrofa.Scrofa 11.1.91) using TopHat version 2.0.14 (Trapnell et al., 2012). The alignment files (in BAM format) were used to calculate raw counts per gene (HTSeq package version 0.9.1) (Anders, Pyl & Huber, 2015). The raw counts per gene were normalized according to gene length and library size using DESeq2 version 1.12.3 (Love, Huber & Anders, 2014) package in Bioconductor (Lawrence et al., 2013) and customized algorithms of the authors (R version 3.4.3). A Principal Component Analysis (PCA) and a Pearson’s correlation analysis between the two biological replicates of TCDD-treated and untreated AhR knock-down cells were performed for logarithmic normalized counts with R statistical software (Wickham, 2009). Differentially expressed genes (DEGs) were identified by comparing the expression levels of genes in AhR knock-down cells treated with TCDD to their respective expression levels in AhR knock-down untreated control cells at each time point (3, 12 and 24 h). The corresponding *P*-values were determined by means of R statistical software using three binominal statistical packages: limma 3.28.6 (Ritchie et al., 2015), edgeR 3.14.0 (Robinson, McCarthy & Smyth, 2010) and DESeq2 for each incubation time. The threshold for the significantly different expression was set at *P-* adjusted <0.05 and log2 fold change (log2FC) ≥ 1.0 or log2FC ≤ −1.0. Only genes that were classified as DEGs by each of the three binominal statistical packages were used for further analysis.

### Functional enrichment analysis

The potential functions of all DEGs were estimated by the Gene Ontology (GO) enrichment analysis (g.Profiler software; *P-* adjusted <0.05) (Reimand et al., 2007). DEGs were classified into three categories of GO database: “biological processes”, “cellular components” and “molecular functions”.

To establish possible interactions between DEGs, the Search Tool for the Retrieval of Interacting Genes (STRING) *S. scrofa* database was used. The STRING is a large database of known and predicted protein interactions that cover more than 1,100 organisms (Franceschini et al., 2013) and includes direct (physical) and indirect (functional) associations. Because the aim of the current study was to identify genes associated with the response of AhR knock-down cells to TCDD, DEGs belonging to the GO “response to stimulus” term were selected to be analyzed in detail by STRING database. The gene interaction network created by STRING is built of nodes and edges, where nodes are defined as individual genes, and edges as interactions between the genes. The number of edges indicate the number of interactions for a particular gene in the network. A protein-protein interaction *P*-value (PPI enrichment *P* value) was calculated, and the cut-off criterion of the combined score was set to >0.7 (high confidence). In addition, the false discovery rate (FDR) test was applied to determine if all DEGs were enriched.

### Real-time PCR

To check the efficacy of the *AhR* gene knock-down in porcine granulosa cells after the siRNA transfection, real-time PCR was performed. The *AhR* gene expression level was determined in C_UT_, C_NEG_ and C_S_ cells after 27 (24 h of transfection + 3 h of cell culture), 36 (24 h of transfection + 12 h of cell culture) and 48 (24 h of transfection + 24 h of cell culture) hours of culture. In addition, real-time PCR was used to validate the results of NGS by analysing the expression of three selected DEGs identified in AhR knock-down granulosa cells treated with TCDD for 3 h. The validation was performed on the same RNA samples which were used for NGS (n= two biological replicates per one time point). The reverse transcription reaction and real-time PCR were performed as previously reported (Sadowska et al., 2017; Ruszkowska et al., 2018). Primers and probes (Thermofisher Scientific, Waltham, MA, USA) for particular genes are presented in Table S2. Glyceraldehyde 3-phosphate dehydrogenase (*GAPDH*) and *β*-actin were used as reference genes.

### Western blotting

To determine the AhR protein abundance in AhR knock-down cells, the cells were cultured in 25 cm^2^ flasks (seeding density: 3 ×10^6^ cells/12 mL culture medium) and transfected with the siRNAs mixture (anti-*AhR* 1 + anti-*AhR* 2 + anti-*AhR* 3; C_S_) and siRNA duplex with an irrelevant sequence (C_NEG_) using Viromer^®^ BLUE as described above. Control cells were cultured without treatments (untransfected cells; C_UT_). Twenty four hours after transfection of the cells with the siRNAs mixture medium was exchanged and the cells were incubated with fresh medium for 3, 12 or 24 h (n = four biological replicates per one time point) to match the experimental design of the TCDD study. After incubation, the cells were designated for total protein isolation. The AhR abundance was determined in C_UT_, C_NEG_ and C_S_ cells after 27 (24 h of transfection + 3 h of cell culture), 36 (24 h of transfection + 12 h of cell culture) and 48 (24 h of transfection + 24 h of cell culture) hours of culture. Protein isolation and western blot analysis were performed as we previously described (Orlowska et al., 2018). Protein samples (15 µg) were separated by SDS-PAGE (9% polyacrylamide gel) and transferred to nitrocellulose membranes (GE Healthcare, Little Chalfont, UK) using the Owl™ VEP-2 Mini Tank Electroblotting System (Thermofisher Scientific) for wet electroblotting (90 mA, 70 min). Following the transfer, the membranes were blocked in TBST containing 5% skimmed milk powder (overnight, 4 °C) and incubated (3 h) with rabbit polyclonal AhR antibodies (ENZO Life Science, New York, NY, USA; diluted in TBST, 1:1,000) or goat polyclonal *β*-actin antibodies (Santa Cruz Biotechnology, Dallas, TX, USA; diluted in TBST, 1:400). Then the membranes were washed with TBST and incubated (1 h, RT) with HRP-conjugated goat anti-rabbit IgG (Santa Cruz Biotechnology, 1:5,000) or donkey anti-goat secondary antibodies (Santa Cruz Biotechnology; diluted in TBST, 1:10,000). Immobilon chemiluminescent HRP substrate (Millipore, 205 Billerica, MA, USA) was employed to visualize immunolabeled bands. The results of the western blotting were quantified by densitometric scanning of immunoblots (n = four replicates for each protein) with Image Studio Lite (version 5.2).

### Statistical analysis

Statistical analysis was performed using Statistica Software (Tulsa, OH, USA). To present the real-time PCR data as arbitrary units of relative expression, gene expression level was normalized to the expression of two reference genes: *GAPDH* and β-actin. This was done by using comparative cycle threshold (C_T_) method and the Quantity Based Active Schematic Estimating (Q-BASE) model (Hellemans et al., 2007). For statistical analysis of the AhR protein abundance, the raw data were normalized using log10 transformation. The densitometric analysis of AhR protein abundance was performed in relation to the reference protein (*β*-actin). The differences in the *AhR* gene expression level as well as in the AhR protein abundance between untransfected cells, cells transfected with irrelevant siRNA sequence and cells transfected with the mixture of three anti-*AhR* siRNAs were evaluated using one-way ANOVA followed by Tukey test. The difference in gene expression level between control and TCDD-treated cells was evaluated using Student’s *t*-test. The level of significance was set at *P* < 0.05 for all analyses. All data are presented as mean ± SEM.

## Results

### The effect of the AhR-targeted siRNAs mixture transfection on the AhR gene expression level and protein abundance in porcine granulosa cells

**Figure 1:**
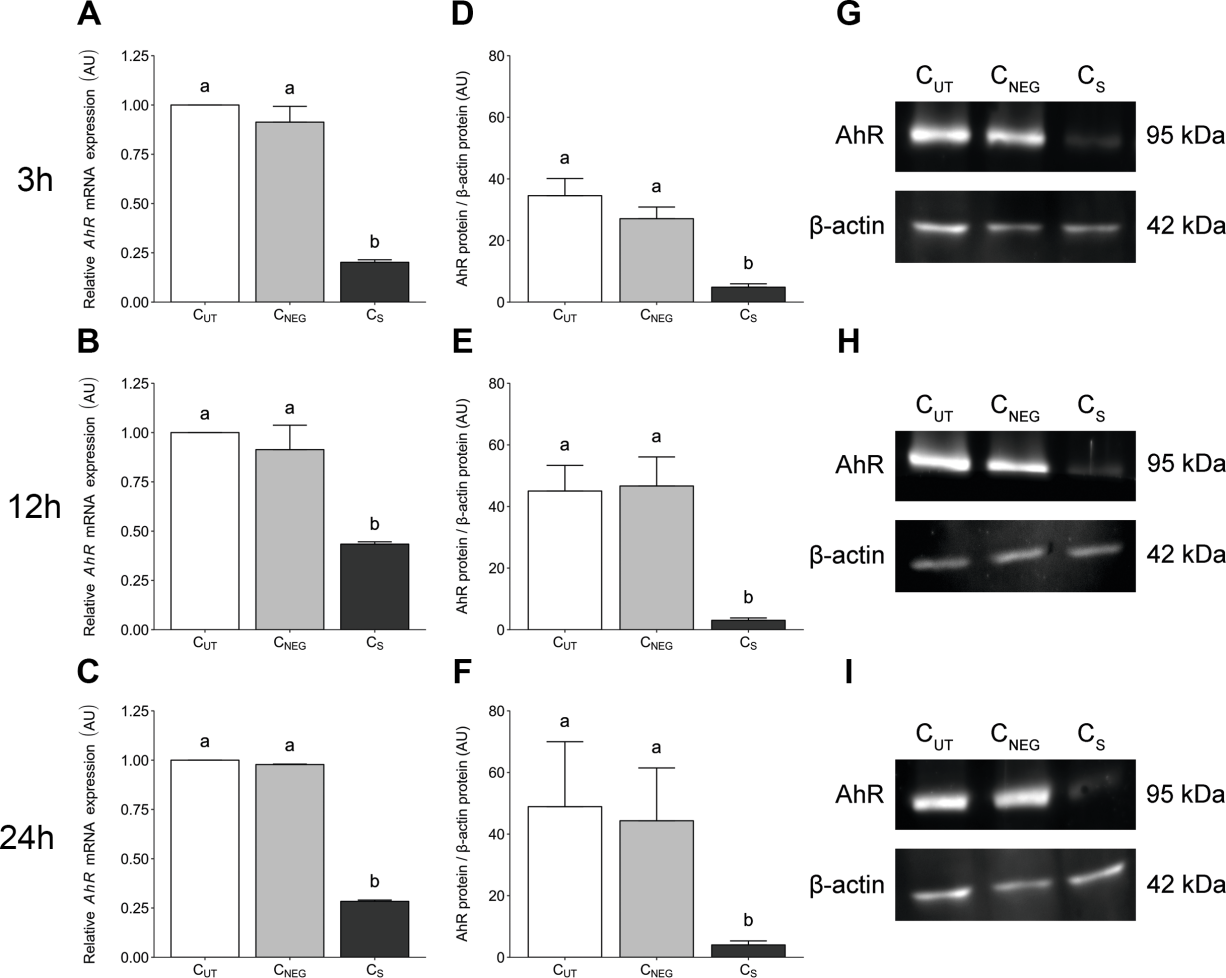
The effects of knock-down of *AhR* gene expression on AhR gene and protein abundance in porcine granulosa cells. The cells were transfected with a mixture of three different siRNAs (24 h) and then cultured for an additional 3, 12 or 24 h. (A–C) *AhR* mRNA expression level was determined by real-time PCR (n = two biological replicates per one time point). Data are expressed as arbitrary units (AU; mean ±  SEM). AhR protein abundance was determined by western blotting (n = four biological replicates per one time point); (D–F) densitometric analysis of the abundance of AhR performed in relation to β-actin. Data are expressed as arbitrary units (AU; mean ± SEM); (G–I) representative immunoblots presented as cropped blots; AhR (95 kDa), β-actin (42 kDa). The whole blots can be found in Supplementary materials (Fig. S1). Statistical analysis was performed using one-way ANOVA followed by Tukey test. “a” and “b” depict statistically significant differences (*P* < 0.05). C_*UT*_: control untreated cells; C_*NEG*_: negative control – cells incubated with siRNA duplex with an irrelevant sequence (nontargeted siRNA); C_*S*_: AhR knock-down cells – cells incubated with the mixture of the three different siRNAs targeting *AhR* (anti-AhR1 + anti-AhR 2 + anti-AhR 3).

*AhR*-targeted siRNA transfection significantly reduced (*P* < 0.05) the expression level of *AhR* gene in porcine granulosa cells at all examined time points (3 (Fig. 1A), 12 (Fig. 1B) and 24 h (Fig. 1C). The time points were selected to match the experimental design of the planned TCDD study. The *AhR* expression level was reduced by 80%, 57% and 72% at 27 (24 h of transfection + 3 h of cell culture), 36 (24 h of transfection + 12 h of cell culture) and 48 (24 h of transfection + 24 h of cell culture) hours of culture, respectively. Nontargeted siRNA transfection (the negative control) had no effect on the *AhR* expression level (*P* >  0.05; Figs. 1A–1C).

*AhR*-targeted siRNA transfection significantly reduced (*P* < 0.05) the AhR protein abundance in porcine granulosa cells cultured for 3 (Fig. 1D), 12 (Fig. 1E) and 24 h (Fig. 1F). No difference was observed in AhR protein abundance between control untransfected cells (C_UT_) and cells transfected with irrelevant siRNA sequence (C_NEG_) (*P* >  0.05). The representative (cropped) blots for AhR and β-actin protein are presented in Figs. 1G–1I. The whole blots can be found in (Fig. S1).

### The effects of TCDD on the transcriptome of AhR knock-down porcine granulosa cells

**Figure 2:**
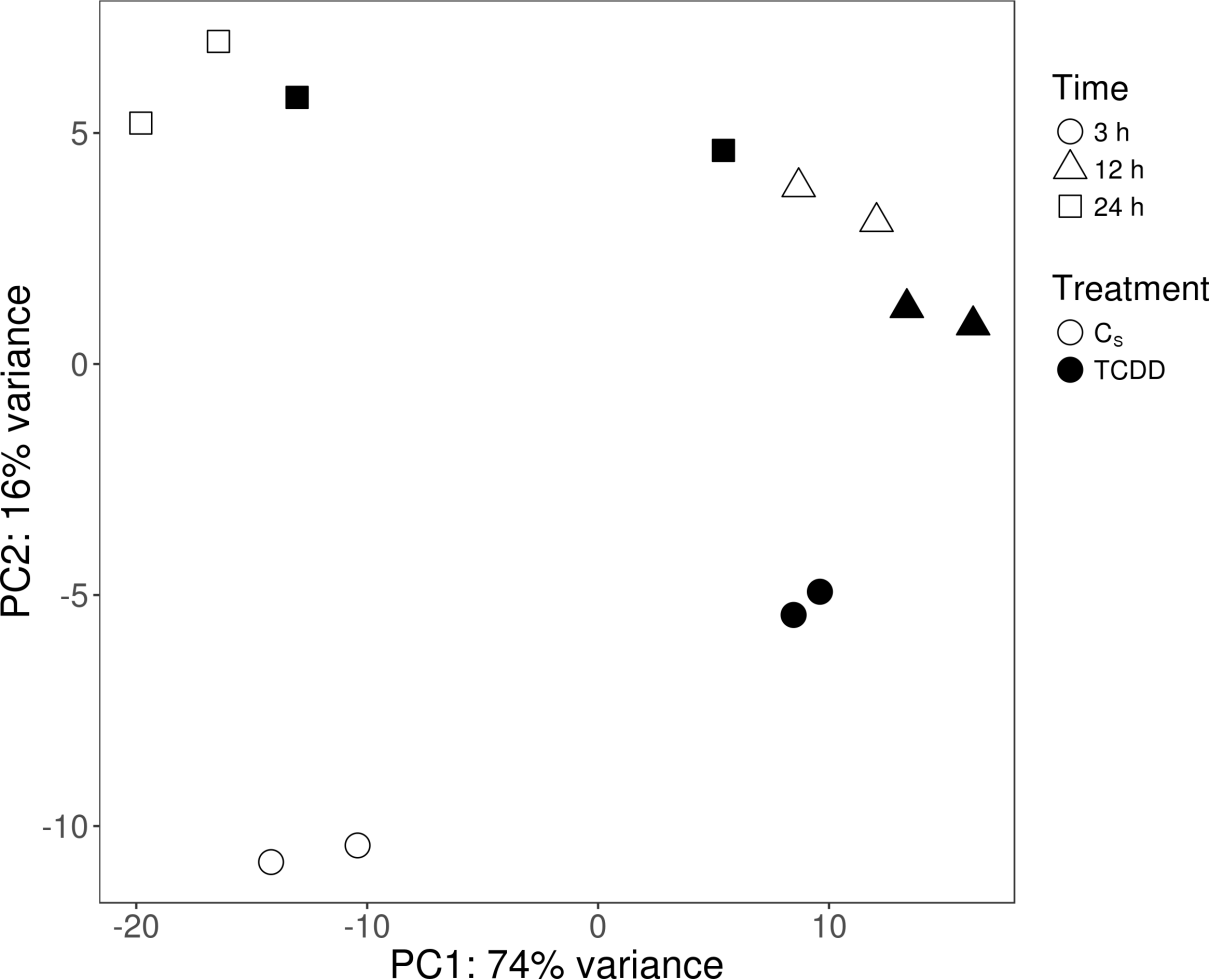
Graphical representation of the first (PC1) and second (PC2) principal components (PC) affecting gene expression pattern of cultured AhR knock-down porcine granulosa cells. C_*S*_: untreated AhR knock-down cells; TCDD: AhR knock-down cells treated with 2,3,7,8-tetrachlorodibenzo-*p*-dioxin (100 nM); 3 h, 12 h, 24 h: hours of cell incubation. The figure was generated with ggplot2 package of R software version 3.4.3 (R Core Team, 2018).

**Figure 3:**
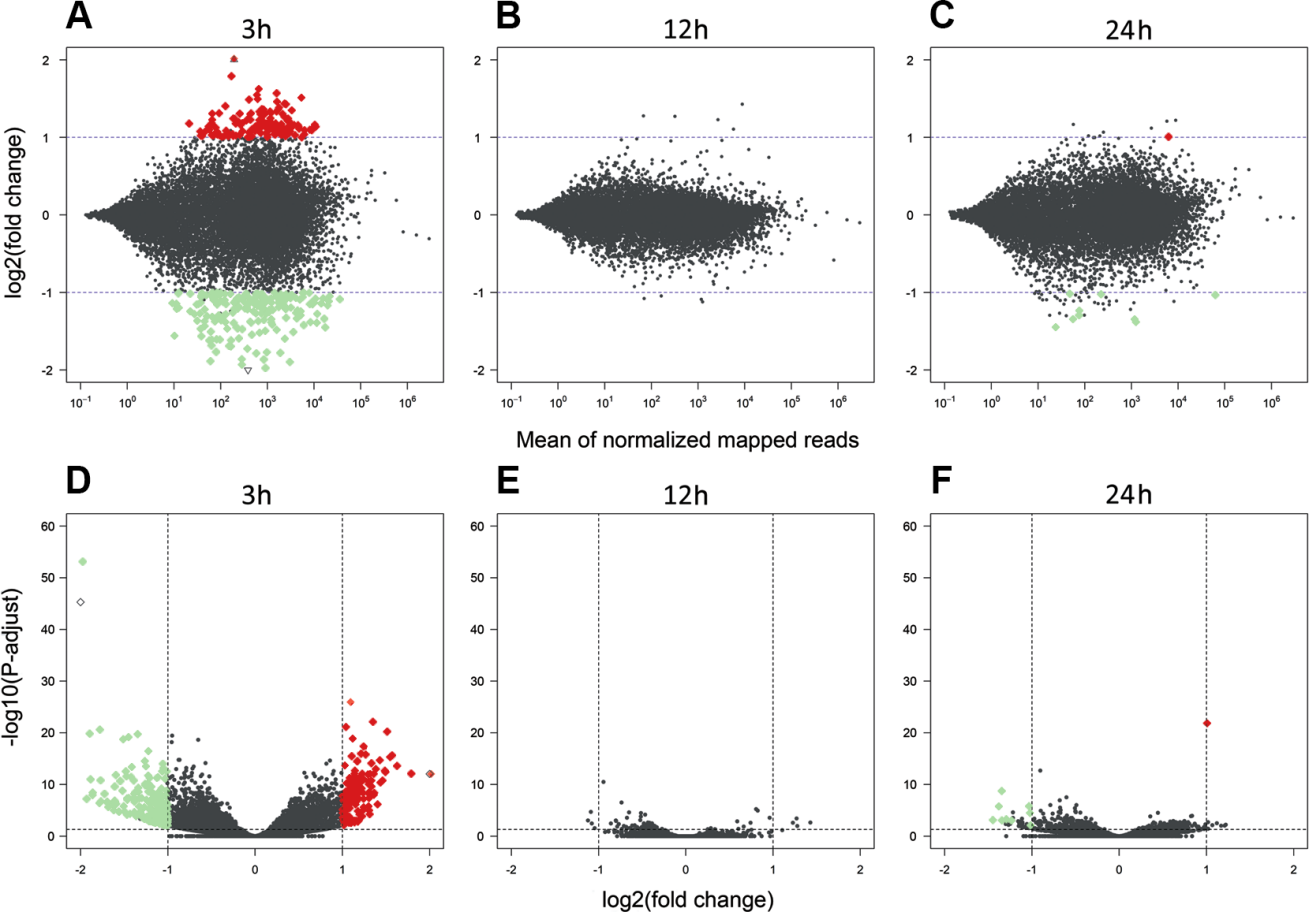
Differentially expressed genes identified in AhR knock-down porcine granulosa cells treated with 2,3,7,8-tetrachlorodibenzo-*p*-dioxin (TCDD). The MA plot (A–C) and Volcano plot (D–F) present differentially expressed genes (DEGs; normalized counts, *P*-adjusted < 0.05 and log2 fold change [log2FC] ≥ 1.0 or log2FC ≤ −1.0) in TCDD-treated AhR knock-down porcine granulosa cells vs. untreated AhR knock-down cells in three different incubation times (3, 12 or 24 h). DEGs are represented by multicolored diamonds, where red color means up-regulated DEGs and green color down-regulated DEGs. The black dots represent all genes that were identified in the cells, and triangles depict expression level values outside the coordinate system. The figure was generated by R software version 3.4.3 (R Core Team, 2018).

The sequencing data from the study have been submitted to the European Nucleotide Archive database (https://www.ebi.ac.uk/ena) under accession number: PRJEB29985. Sequencing of the AhR knock-down porcine granulosa cell transcriptome provided 779.9 million raw reads, ranging from 51.6 million to 79.5 million per sample. After removing reads containing adapters and low-quality reads (reads length <  90 bp; Phred score Q <  20), the remaining 746.6 million high-quality reads (48.5 to 76.9 million per sample) were mapped to the Ensembl porcine genome with GTF annotation file (Sus Scrofa.Scrofa 11.1.91). The number of reads aligned to the reference genome ranged from 44.5 to 69.5 million per sample, and an average of 96% of these reads were mapped to unique locations. The total number of genes expressed in AhR knock-down granulosa cells of all examined samples ranged from 15,100 to 15,913 (Table S3). The Pearson correlation coefficients (*P* <0.0001) between the two biological replicates of TCDD-treated and untreated AhR knock-down granulosa cells collected at each time point ranged between 0.971 and 0.999 (Table S4). The results of the PCA revealed that the most marked differences in gene expression pattern between control and TCDD-treated cells were found after 3 h of the treatment. In contrast, the least differences were observed after 12 h. Moreover, genes with expression significantly affected by TCDD after 3-h culture were clustered separately from those of 12- and 24-h cultures (Fig. 2). The MA and Volcano plots present the significant changes (*P*-adjusted <0.05, log2FC ≥ 1.0 or log2FC ≤ −1.0) in gene expression profiles of AhR knock-down granulosa cells treated with TCDD compared to AhR knock-down control cells (Fig. 3).

**Figure 4:**
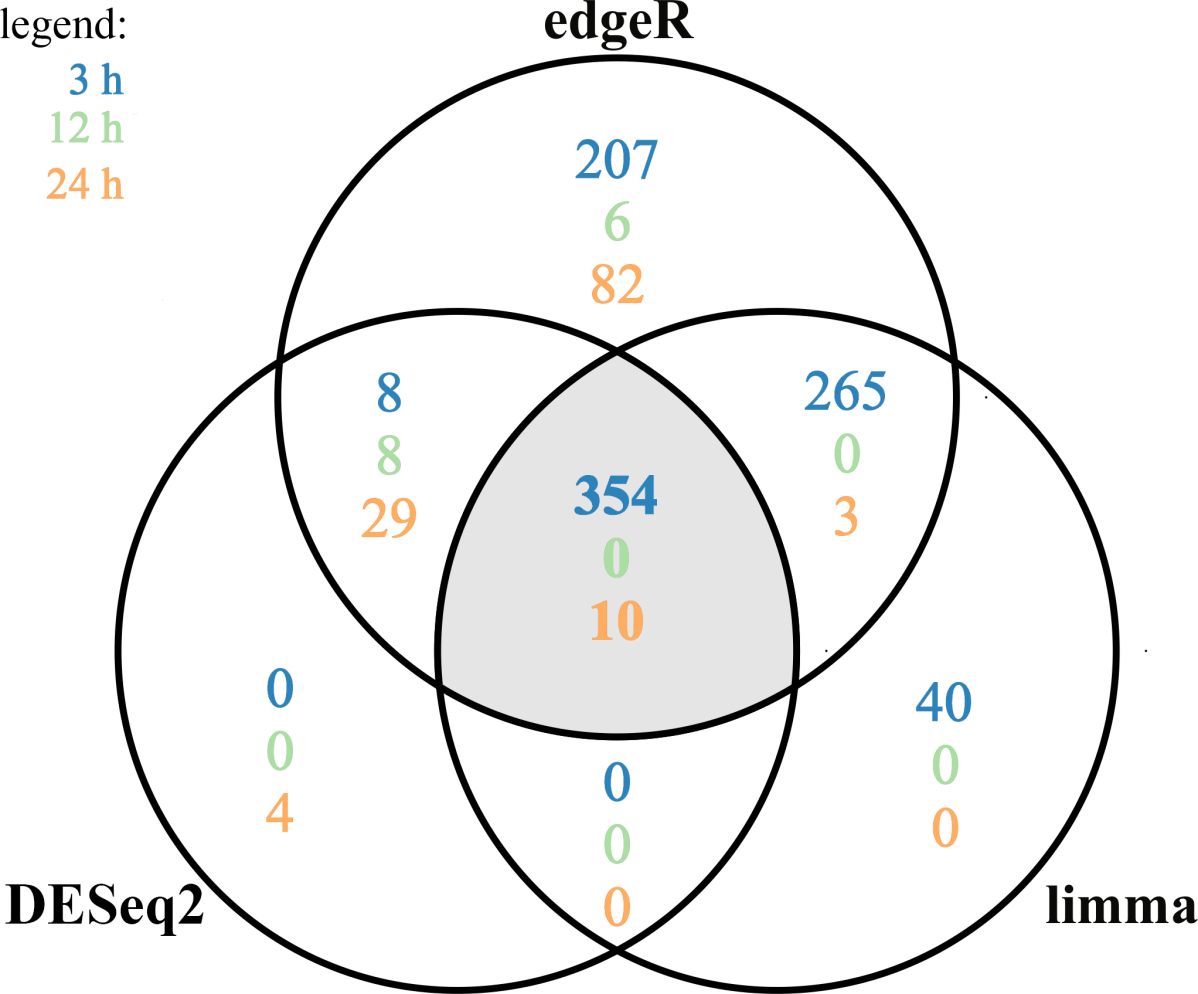
Differentially expressed genes (DEGs) identified in AhR knock-down porcine granulosa cells treated with 2,3,7,8-tetrachlorodibenzo-*p*-dioxin (TCDD) for 3, 12 or 24 h presented as Venn diagram. Reads mapped to porcine genome were next analyzed by means of three different binominal statistical packages: edgeR, DESeq2 and limma. Overlapping circles (grey field) present DEGs that are common for the individual statistical packages. The results were considered statistically significant at *P*-adjusted < 0.05 and log2 fold change (log2FC) ≥ 1.0 or log2FC ≤ −1.0. The figure was generated by R software version 3.4.3.

**Figure 5:**
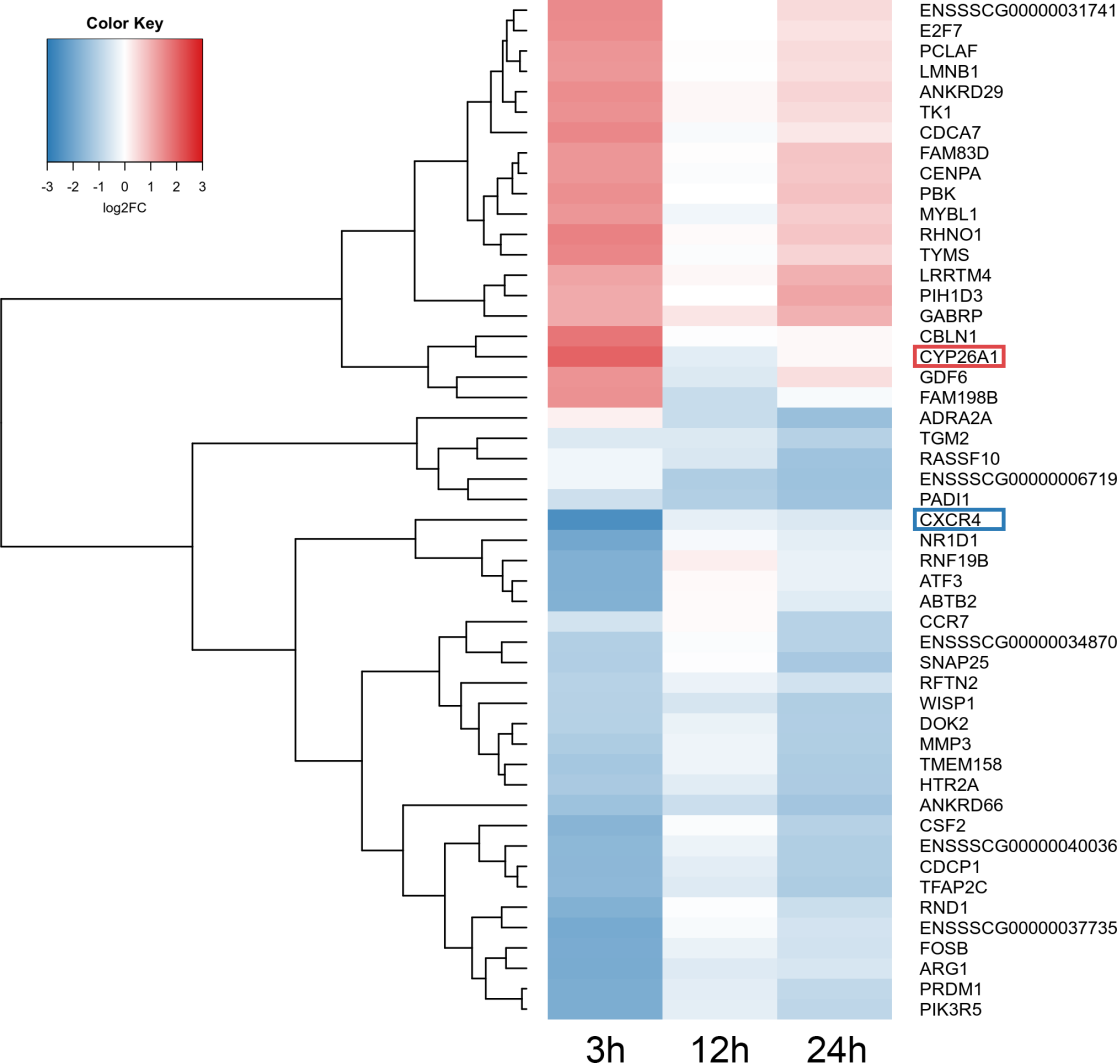
Heatmap of the top 50 differentially expressed genes (DEGs) demonstrated in AhR knock-down porcine granulosa cells treated with TCDD for 3, 12 or 24 h. The results were considered statistically significant at *P*-adjusted < 0.05 and log2 fold change (log2FC) ≥ 1.0 or log2FC ≤ −1.0. Red blocks represent up- and blue blocks down-regulated genes. The color scale of the heatmap shows the expression level where the brightest blue stands for −3.0 log2FC and the brightest red stands for 3.0 log2FC. DEGs with the most up-regulated (*CYP26A1*; log2FC = 2.016) and the most down-regulated (*CXCR4*; log2FC = −2.542) expression level were framed. TCDD: 2,3,7,8-tetrachlorodibenzo-*p*-dioxin. The figure was generated by gplots package of R software version 3.4.3 (R Core Team, 2018).

A total of 360 DEGs were identified in the current study (Table S5). We found 354 (148 up- and 206 down-regulated) DEGs after 3 h of TCDD treatment and only 10 (1 up- and 9 down-regulated) DEGs after 24 h of the treatment. No DEGs were found after 12 h of the TCDD treatment (Fig. 4). Among all DEGs, four genes (odontogenesis associated phosphoprotein [*ODAPH*]; ankyrin repeat domain 66 [*ANKRD66*]; synaptosome associated protein 25 [*SNAP25*]; gamma-aminobutyric acid type A receptor pi subunit [*GABRP*]) were identified at two time points, i.e., after 3 and 24 h of TCDD treatment. The log2FC value for DEGs ranged from -2.54 (CXC chemokine receptor type 4; *CXCR4*) to +2.02 (cytochrome P450, family 26, subfamily A, member 1; *CYP26A1*). TCDD did not affect *AhR* as well as *CYP1A1* gene expression at any of the examined time points. The expression profile of the top 50 DEGs (i.e., DEGs with the highest log2FC value) is presented in Fig. 5.

### Functional enrichment analysis of DEGs

**Figure 6:**
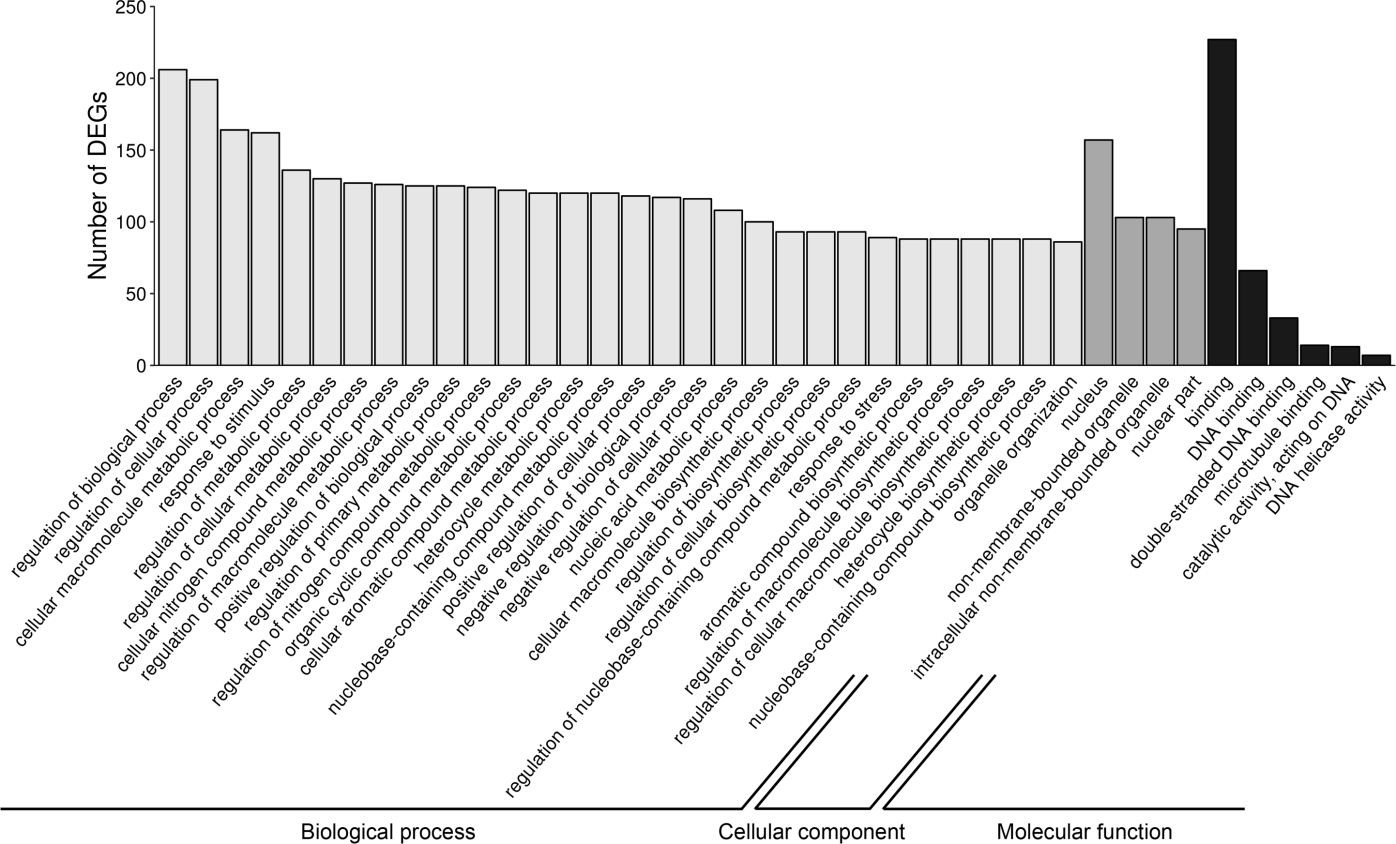
Gene Ontology (GO) analysis of differentially expressed genes (DEGs) identified in AhR knock-down porcine granulosa cells treated with 2,3,7,8-tetrachlorodibenzo-*p*-dioxin (TCDD) for 3, 12 or 24 h. 324 out of 360 DEGs were classified into three categories of the GO database (“biological processes”, “cellular components” and “molecular function”). The figure presents only the subcategories with the highest number of the ascribed DEGs. GO enrichment analysis was performed using g.Profiler software with P-adjust threshold 0.05. The figure was generated by ggplot2 package of R software version 3.4.3 (R Core Team, 2018).

To investigate the possible significance of the identified DEGs in the AhR knock-down porcine granulosal response to TCDD, the genes were classified to three main categories (“biological processes”, “cellular components” and “molecular function”) according to GO database as described in Materials & Methods. Three hundred twenty four out of 360 DEGs were assigned to 172 GO terms (*P-* adjusted <  0.05); within these GO terms 145 were ascribed to “biological processes”, 21 to “cellular components” and 6 to “molecular function” category (Table S6). The most DEGs of “biological processes” category were allocated to “regulation of biological process” (GO:0050789; 206 DEGs), “regulation of cellular process” (GO:0050794; 199 DEGs), “cellular macromolecule metabolic process” (GO:0044260; 164 DEGs) and “response to stimulus” (GO:0050896; 162 DEGs) (Fig. 6). Most of the genes ascribed to the “cellular component” category were annotated to “nucleus” (GO:0005634; 157 DEGs), “non-membrane-bounded organelle” (GO:0043228; 103 DEGs) and “intracellular non-membrane-bounded organelle” (GO:0043232; 103 DEGs). The most enriched GO term in the “molecular function” category was “binding” (GO:0005488; 227 DEGs) (Fig. 6).

**Figure 7:**
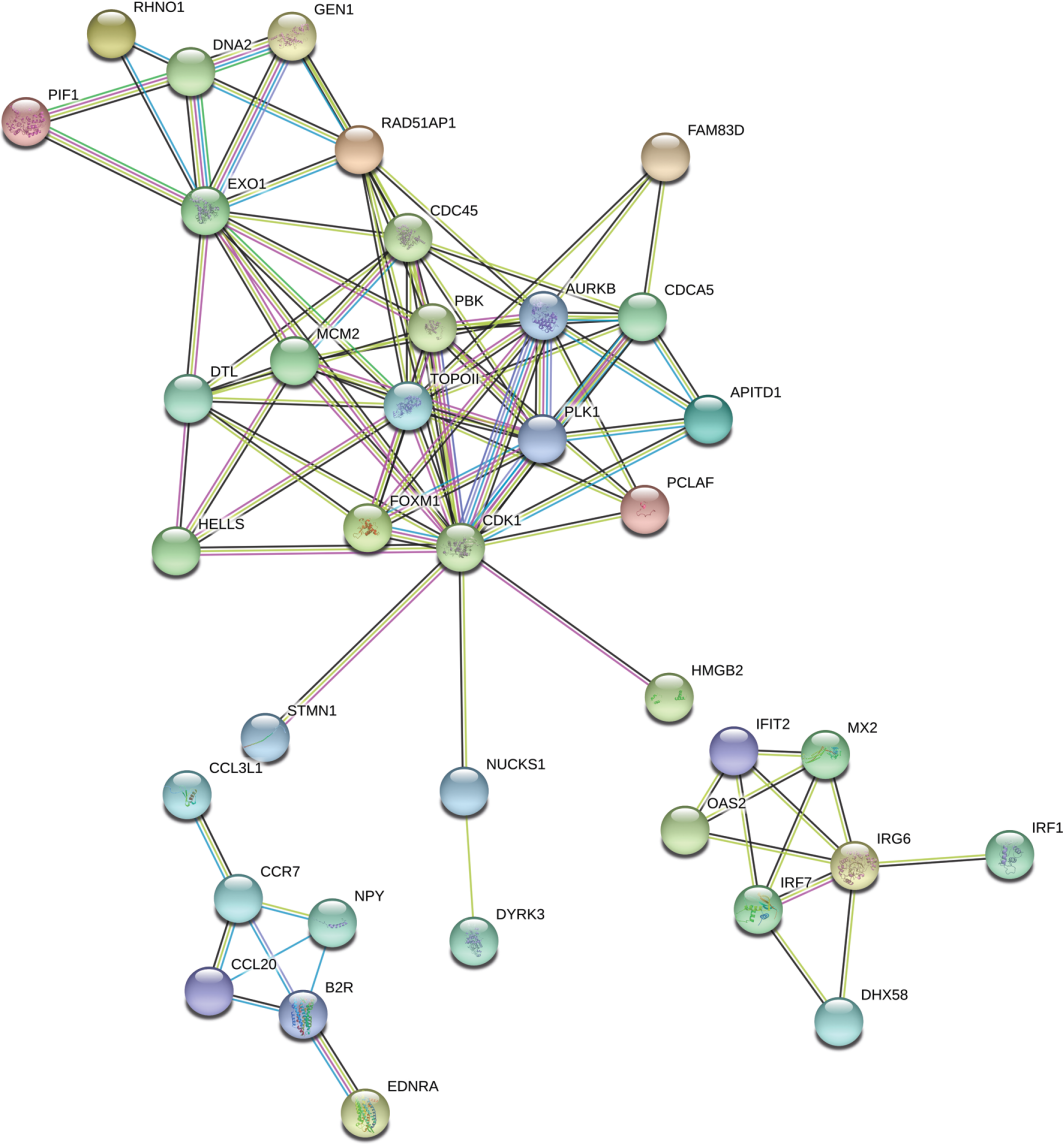
Interaction network of differentially expressed genes (DEGs) identified in AhR knock-down porcine granulosa cells treated with 2,3,7,8-tetrachlorodibenzo-*p*-dioxin (TCDD) for 3, 12 or 24 h. The network was generated by STRING (confidence score: 0.7) using the DEGs (*P*-adjusted < 0.05 and log2 fold change [log2FC] ≥ 1.0 or log2FC ≤ 1.0) belonging to the GO “response to stimulus” term (GO:0050896). Enrichment *P*-value: 1.0 ×10^−16^.

The “response to stimulus” was one of the most enriched GO term containing 162 DEGs. Functional classifications of these genes performed with the use of STRING produced a gene interaction network with 119 nodes and 105 edges (PPI enrichment *P* - value: 1.0 × 10^−16^; Fig. 7). The network included genes related to: (i) cell cycle regulation (cell division cycle 45 (*CDC45*), minichromosome maintenance complex component 2 (*MCM2*), RAD51 associated protein 1 (*RAD51AP1*), cyclin-dependent kinase 1 (*CDK1*), polo like kinase 1 (*PLK1*), aurora kinase B (*AURKB*) and DNA topoisomerase 2-alpha (*TOPOII*)), (ii) interferon signaling pathway (interferon induced protein with tetratricopeptide repeats 2 (*IFIT2*), 2′-5′-oligoadenylate synthetase 2 (*OAS2*), interferon regulatory factor 1 (*IRF1*), interferon regulatory factor 7 (*IRF7*), inflammatory response protein 6 (*RSAD2*)) and (iii) cytokine-cytokine receptor interaction (C-C motif chemokine ligand 20 (*CCL20*), C-C chemokine receptor type 7 precursor (*CCR7*)). Among the most interacting nodes were *CDK1* (15 edges), *TOPOII* (13 edges), *CDC45* (10 edges) and *IRG6* (6 edges).

### Validation of NGS data by real-time PCR

**Figure 8:**
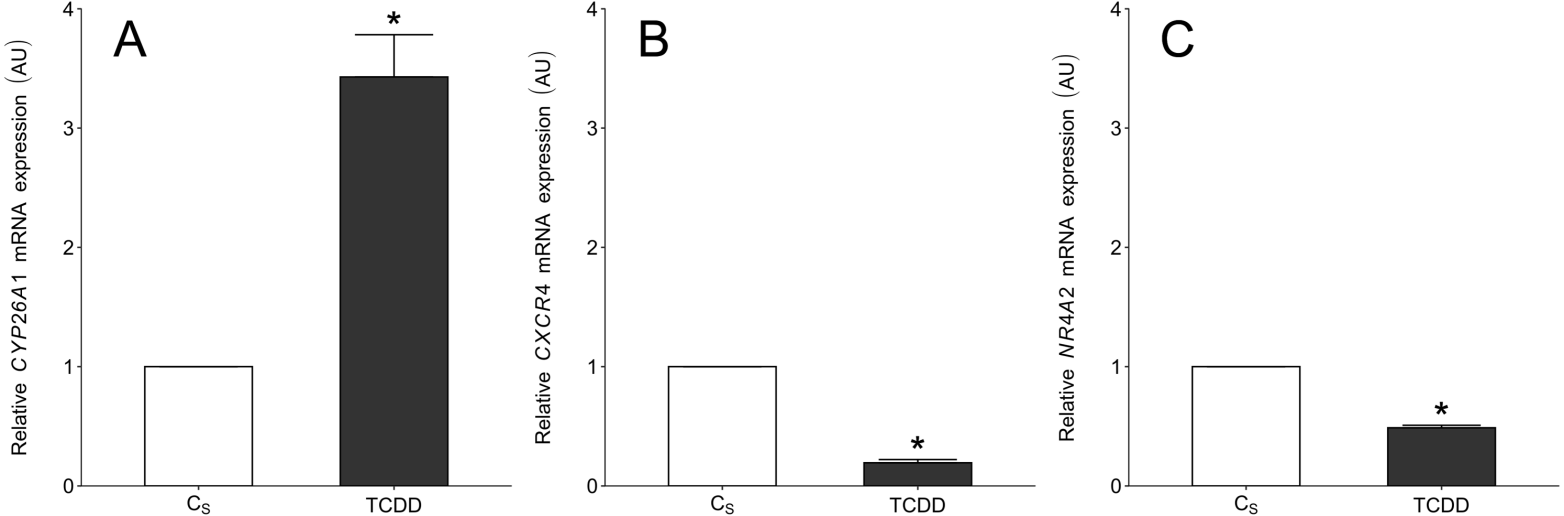
Real-time PCR validation of three selected differentially expressed genes which were identified in TCDD-treated AhR knock-down porcine granulosa cells (treated vs. untreated cells) by NGS. To validate the NGS results, CYP26A1 (A), CXCR4 (B) and NR4A2 (C) were chosen for real-time PCR. The validation was performed on the same RNA samples which were used for NGS (n = two biological replicates per one time point). Data were expressed as arbitrary units (AU; mean ± SEM). Statistical analysis was performed using t-student test. Asterisks designate statistical differences (*P* < 0.05). AU: arbitrary units; C_*S*_: untreated AhR knock-down cells; TCDD: AhR knock-down cells treated with 2,3,7,8-tetrachlorodibenzo-*p*-dioxin.

To validate the NGS results, *CYP26A1*, *CXCR4* and *NR4A2* (nuclear receptor subfamily 4, group A, member 2) were chosen for real-time PCR. The first two DEGs were selected based on their log2FC value (top up- and down-regulated genes) as well as their potential importance for granulosa cell functions. The *NR4A2* was selected because the gene was also identified in our previous study concerning the TCDD effects on intact porcine granulosa cells (Sadowska et al., 2017). The expression patterns of these three DEGs obtained by real-time PCR were in agreement with NGS results (Fig. 8).

## Discussion

In the current study, the NGS approach was used to examine changes in the transcriptome of AhR knock-down AVG-16 cells exposed to TCDD. The AVG-16 cell line was derived from granulosa cells of medium porcine follicles (Horisberger, 2006), and the cells were reported to exhibit AhR expression, both at mRNA and protein level (Sadowska et al., 2015). Previously, we employed the intact (untransfected) AVG-16 cells to study the impact of TCDD on the gene (Sadowska et al., 2017; Nynca et al., 2019) and lncRNA expression profiles (Ruszkowska et al., 2018) as well as on the cell proteome (Orlowska et al., 2018).

The AhR gene and protein abundance in the cultured AhR knock-down AVG-16 cells was significantly reduced in comparison to control untransfected cells. The effective transfection of the cells was achieved with the use of Viromer Blue transfection reagent and a mixture of three different siRNAs. The degree of the AhR knock-down at the level of gene expression was on average 70% which is a typical value for this method (Hood, 2004).

The changes in the gene expression profile of AhR knock-down granulosa cells were examined after 3, 12 and 24 h of TCDD treatment. Due to the fact that the expression of particular genes reach a peak or a nadir at different times after a treatment, a one-time-point-study does not allow for ascertaining some important changes in gene expression as well as does not allow for proper investigation of changes overtime in the transcriptomic profile (Nynca et al., 2019). In consequence, a total of 360 DEGs was identified in AhR knock-down cells treated with TCDD for 3, 12 and 24 h. Similarly to previously reported TCDD-induced changes in gene expression level of intact porcine granulosa cells (Sadowska et al., 2017), most of the presently identified DEGs (354 out of 360) were detected after three hours of TCDD treatment. Interestingly, the expression level of most lncRNAs recognized in our recent study performed on intact granulosa cells was also significantly altered after three hours of the dioxin treatment (Ruszkowska et al., 2018). Moreover, we have recently demonstrated, with the use of multifactorial analysis (where TCDD and time were the factors), that TCDD-induced changes in the intact porcine granulosa cell transcriptome were especially pronounced during the first 12 h of the treatment (Nynca et al., 2019). The results obtained in the current study conducted out on AhR knock-down cells together with those reported previously clearly indicate that TCDD effects on porcine granulosa cells are time-dependent. It seems that regardless of the AhR status of the cells, TCDD affects the cell transcriptome predominately during the early stages of its action.

Out of 360 DEGs affected by TCDD in AhR knock-down cells, 354 DEGs were detected after 3 h of TCDD treatment. No DEGs were found after 12 h and only 10 DEGs were identified after 24 h of the treatment. Similar, in the kidney or liver of AhR^−∕−^ mice examined after 24 h (Boutros et al., 2009) or 19 h (Tijet et al., 2006) of TCDD treatment, the expression of 5 or 32 genes was significantly altered by TCDD, respectively. It cannot be excluded that considerably more genes with expression affected by TCDD would be found if the AhR^−∕−^ mice tissues had been examined earlier i.e., 2-4 h after the initiation of TCDD treatment.

It is puzzling why the expression level of so many genes was significantly affected after 3 h – but not after 12 and 24 h – of TCDD treatment of the AhR knock-down cells. Apparently, the TCDD stimulus was so important for the cells that it provoked their massive response, involving genes enriched in such GO terms as “regulation of metabolic process”, “binding”, “nucleus” or “response to stress”. The later response of the AhR knock-down cells (12–24 h) to the TCDD was considerably less abundant (0–10 genes). Experiments performed on AhR^−∕−^ mice which also exhibited only a few genes affected by TCDD (Tijet et al., 2006; Boutros et al., 2009) support our results. We were not able, however, to find any information that would help to shed some light on this striking issue. AhR downregulation, characteristic for TCDD action (Pollenz, 2002), does not seem to be responsible for this phenomenon since TCDD did not affect *AhR* gene expression at any of the examined time points in the present study. It appears that the modest reaction of AhR knock-down cells demonstrated during the later stages of the dioxin action might be helpful in explaining the previously reported resistance of AhR knock-out mice and rats to TCDD (Harrill et al., 2016).

The large number of DEGs found in AhR knock-down granulosa cells treated with TCDD clearly suggests that this dioxin may affect its target tissues in an AhR-independent manner. Receptor Ah is considered to be the main mediator of TCDD intracellular action (Denison et al., 2011) and none of the alternative mechanisms are well documented. Although it is commonly believed that xenoestrogens may act *via* the genomic activation of various steroid hormone receptors (e.g., estrogen receptors (ER)) or other nuclear receptors (e.g., pregnane X (PXR) and constitutive androstane (CAR) receptors) (Wang et al., 2012; Xu et al., 2017), there is no clear evidence – with the exception of ER (Swedenborg & Pongratz, 2010) – indicating their involvement in the TCDD signaling pathway. TCDD and other xenoestrogens are also known to be potent activators of various nongenomic responses (Matsumura, 2011; Xu et al., 2017). However, the presence of AhR-independent TCDD signaling pathways in porcine granulosa cells has yet to be proven. On the other hand, one can argue that TCDD might act *via* the activation of the remaining, not knock-down AhR (<14% relatively to untransfected cells). This mode of action, however, does not seem very likely since no DEGs or very few DEGs were identified after 12–24 h of TCDD treatment.

The recognition of genes associated with the early response in AhR knock-down porcine granulosa cells is therefore very important. In the current study, the TCDD treatment of AhR knock-down cells did not affect the classical AhR gene battery i.e., genes encoding xenobiotic-metabolizing enzymes, such as cytochrome P450, family 1, member A1 (*CYP1A1*), member A2 (*CYP1A2*) or member B1 (*CYP1B1*). Similarly, TCDD did not significantly affect *CYP1A1* gene expression in the liver of AhR ^−∕−^ mice (Tijet et al., 2006). Previously, we demonstrated that in the intact porcine granulosa cells TCDD induced the transcription of *CYP1A1* (Sadowska et al., 2017) and increased CYP1A1 activity (*T Molcan, 2018, unpublished data*). It seems that, in AhR knock-down cells, TCDD was able to activate neither the classic AhR/ARNT pathway nor any other pathway capable of increasing the *CYP1A1* expression. It should be also emphasized that the failure of TCDD to induce the expression of *CYP1A1* in the AhR knock-down cells additionally confirms the successful knock-down of AhR in porcine granulosa cells.

*CYP26A1* was the most up-regulated gene in the AhR knock-down porcine granulosa cells treated with TCDD. *CYP26A1* is known to encode the enzyme implicated in the oxidative metabolism of retinoic acid (RA) - the active metabolite of vitamin A (Ross & Zolfaghari, 2011). Vitamin A plays a vital role in cell growth and differentiation, it is also important for reproductive processes (Clagett-Dame & Knutson, 2011). It was demonstrated that TCDD – acting probably *via* the AhR pathway – disturbs retinoid homeostasis in different species, including guinea pigs, mice, hamsters and rats (Rolland, 2000; Nilsson & Håkansson, 2002; Fletcher et al., 2005). Such perturbation of retinoid levels led to defective reproduction and developmental abnormalities in humans and animals (Nilsson & Håkansson, 2002). It is possible that TCDD, regardless of the AhR status of the cell, may affect the metabolism of vitamin A and retinoid-associated processes.

*CXCR4* was the most down-regulated gene in the AhR knock-down granulosa cells treated with TCDD. CXCR4, a G protein-coupled receptor for chemokine CXC motif ligand 12 (CXCL12), was reported to be involved in follicular development and oocyte maturation (Sayasith & Sirois, 2014; Choi et al., 2017; Luo et al., 2017; Zhang et al., 2018). Zhang et al. (2018) demonstrated that inhibition of CXCR4 repressed the expression of hyaluronan synthase 2 (*HAS2*) and decreased cumulus expansion in sheep. The *HAS2* gene is responsible for synthesis of hyaluronic acid which, in turn, is tightly associated with cumulus expansion, a process important for proper ovulation and fertilization. It was reported that TCDD can impinge on female fertility by preventing ovulation in rats (Petroff et al., 2001). The inhibitory effect of TCDD on *CXCR4* expression in the examined cells clearly supports this notion.

STRING-based classification of DEGs demonstrated that the most significant regulatory networks were related to cell cycle regulation, interferon signaling pathway and cytokine-cytokine receptor interaction. Among DEGs classified to the cell cycle regulation network we found genes encoding proteins required to initiate DNA replication (*CDC45* and *MCM2*) (Aparicio, Ibarra & Méndez, 2006), DNA repair protein (*RAD51AP1*) (Pires, Sung & Wiese, 2017), an enzyme essential for proper segregation of chromosomes (*TOP2A*) (Porter & Farr, 2004) and kinases regulating the cell division (*CDK1, PLK1 and AURKB*) (Salaun, Rannou & Prigent, 2008). In the current study, the expression of all these genes was up-regulated by TCDD. Recently, it was demonstrated in intact porcine granulosa cells that TCDD also affected the abundance of genes (Sadowska et al., 2017) and proteins (Orlowska et al., 2018) involved in cell proliferation and differentiation. These findings suggest that TCDD may disturb the cell cycle progression regardless of the AhR status of the granulosa cells. Interestingly, the genes expression of which was up-regulated by TCDD in the current study were also reported to be over-expressed in several types of cancer cells (Das et al., 2014; Köhler et al., 2016; Tang et al., 2017; Chudasama et al., 2018; Gou et al., 2018; Jeong et al., 2018; Pei, Yin & Liu, 2018). It is possible that the exposure of AhR knock-down porcine granulosa cells to TCDD may lead to genomic instability of cells. This hypothesis, however, needs to be tested.

We found that 13 differentially expressed genes (DEGs; Table S7) identified in the current study were also recognized as DEGs in our previous study on TCDD effects in intact porcine granulosa cells (Sadowska et al., 2017). These two experiments were performed on the same cell type (AVG-16 cells), both employed NGS and had the same experimental design. The common genes include DNA-binding protein inhibitor (*ID1*), the orphan receptor family members: nuclear receptor subfamily 4, group A, member 2 and 3 (*NR4A2; NR4A3*), ankyrin repeat domain 1 (*ANKRD1*) and MAF bZIP transcription factor F (*MafF*). The five genes encode transcription factors implicated in cell growth and differentiation, inflammation, stress response, apoptosis and oncogenesis. Their expression was reported to be regulated by well-known modulators of ovarian functions such as gonadotropins, prostaglandins, growth factors, cytokines and xenobiotics. ID1 was suggested to control angiogenesis associated with luteinization (Leo, Pisarska & Hsueh, 2001). NR4A2 was found to be involved in the regulation of steroidogenic enzyme expression as well as gonadotropin secretion. The expression of *NR4A2* and *NR4A3*, in turn, was reported to be induced in granulosa cells following the LH preovulatory surge (Bertolin et al., 2010). ANKRD1, a target of TGF-β/Wnt signaling, appears to be linked with apoptosis and oxidative stress (Nivet et al., 2013; Lei et al., 2015). Finally, MafF affected genes encoding proteins responsible for xenobiotic metabolism and antioxidation (Kannan, Solovieva & Blank, 2012). It is of interest that *MafF* was found to be located in the antisense strand of the lncRNA (TCONS_00038918), the expression of which was affected by TCDD in the intact porcine granulosa cells (Ruszkowska et al., 2018). The 13 DEGs common for AhR knock-down and intact porcine granulosa cells were usually (9 out of 13 DEGs) inversely affected by TCDD (up- vs. down-regulation) in the two examined cell types. Similarly, Guo et al. (2004) demonstrated that the expression of most genes mutual for wild type and AhR knock-out cultured mouse aorta smooth muscle cells responded to TCDD in an opposite manner. These results suggest that, regardless of the AhR status of the cells, TCDD may employ the same signaling molecules, using them, however, in a different configuration. In addition, the large number of DEGs that differ between the current study (AhR knock-down cells; 347 [360-13] DEGs) and the study performed on intact granulosa cells (128 [141-13] DEGs) also confirms the concept that TCDD may affect the cells in an AhR-independent manner.

## Conclusions

To the best of our knowledge, this is the first study employing NGS to examine TCDD-induced changes in the transcriptome of AhR knock-down cells. In AhR knock-down porcine granulosa cells, TCDD influenced the expression of 360 genes, including those involved in the metabolism of vitamin A (*CYP26A1*), follicular development and oocyte maturation (*CXCR4),* proliferation and differentiation (*CDC45, RAD51AP1, TOP2A, CDK1, PLK1*) as well as inflammation, stress response, apoptosis and oncogenesis (*ID1*, *ANKRD1*, *NR4A2*, *NR4A3*, *MafF*). The three-time point study enabled us to demonstrate that TCDD-induced changes in the transcriptome of AhR knock-down porcine granulosa cells were especially pronounced during the early stages (3 h, 354 DEGs) of the dioxin action. The identification of DEGs common for AhR knock-down and intact cells treated with TCDD indicated that TCDD, dependent on the AhR status of the cells, may use the same signaling components, which may be however, differently entwined in the signaling network. The obtained results revealed the ability of TCDD to alter gene expression in an AhR-independent manner and indicated molecules that may potentially be involved in such action. This study offers broader insight into the mechanism of TCDD action and provides a foundation for future research focused on molecular effects exerted by TCDD.

##  Supplemental Information

10.7717/peerj.8371/supp-1 Figure S1 The effects of knock-down of *AhR* gene expression on AhR protein abundance in porcine granulosa cells The cells were transfected with three different siRNAs (24 h) and then cultured for an additional 3, 12 or 24 h. AhR protein abundance was determined by western blotting (*n* = 4 biological replicates per one time point). Unedited blots for Figure 1C in the manuscript; representative immunoblots showed in Figure 1C were cropped to white-line boxes. C_*UT*_: control untreated cells; C_*NEG*_: negative control –cells incubated with siRNA duplex with an irrelevant sequence (nontargeted siRNA); C_*S*_: AhR knock-down cells incubated with the mixture of the three different siRNAs targeting *AhR* (anti-AhR1 + anti-AhR 2 + anti-AhR 3).

10.7717/peerj.8371/supp-2 Table S1 The sequences of siRNAs used to knock-down porcine aryl hydrocarbon receptor (AhR) gene

10.7717/peerj.8371/supp-3 Table S2 Primers and probes used for real-time PCR

10.7717/peerj.8371/supp-4 Table S3 Summary of datasets used in the study

10.7717/peerj.8371/supp-5 Table S5 Pearson’s correlation coefficients between two biological replicates of TCDD-treated and untreated AhR knock-down porcine granulosa cells cultured for 3, 12 and 24 hours

10.7717/peerj.8371/supp-6 Table S5 Differentially expressed genes (DEGs) identified in AhR knock-down porcine granulosa cells after 3, 12 or 24 hours of 2,3,7,8-tetrachlorodibenzo-p-dioxin (TCDD) treatment

10.7717/peerj.8371/supp-7 Table S6 Functional enrichment analysis of differentially expressed genes (DEGs) identified in AhR knock-down porcine granulosa cells after 3, 12 or 24 h of 2,3,7,8-tetrachlorodibenzo-p-dioxin (TCDD) treatment

10.7717/peerj.8371/supp-8 Table S7 Differentially expressed genes indentified both in the current study and in our previous study devoted to examining the TCDD effects in intact porcine granulosa (Sadowska et al., 2017)

## Additional Information and Declarations

### Data Availability

The following information was supplied regarding data availability: The datasets analyzed during the current study are available in the European Nucleotide Archive database: PRJEB29985.
