# Supplementary material for: The effects of 2,3,7,8-tetrachlorodibenzo-p-dioxin (TCDD) on the transcriptome of aryl hydrocarbon receptor (AhR) knock-down porcine granulosa cells
Source: PeerJ. 2020 Jan 22;8:e8371. doi: 10.7717/peerj.8371 (PMC6982409; doi:10.7717/peerj.8371)
Supplement: Figure S1 — The cells were transfected with three different siRNAs (24 h) and then cultured for an additional 3, 12 or 24 h. AhR protein abundance was determined by western blotting (n = 4 biological replicates per one time point). Unedited blots for Figure 1C in the manuscript; representative immunoblots showed in Figure 1C were cropped to white-line boxes. CUT: control untreated cells; CNEG: negative control –cells incubated with siRNA duplex with an irrelevant sequence (nontargeted siRNA); CS: AhR knock-down cells incubated with the mixture of the three different siRNAs targeting AhR (anti-AhR1 + anti-AhR 2 + anti-AhR 3). [file peerj-08-8371-s001.pdf]

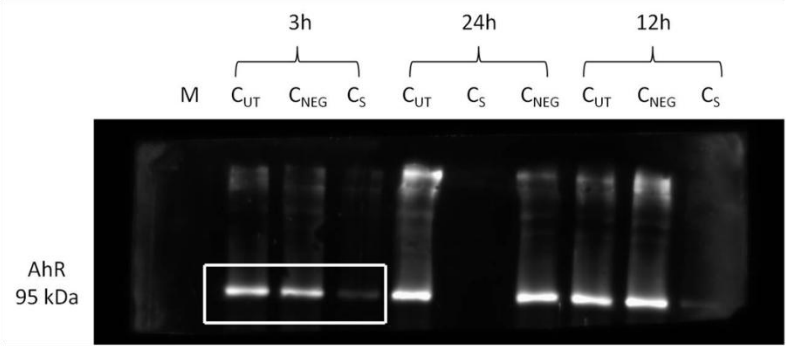

Full length Western blots corresponding to the panels shown in Figure 1C (3h).

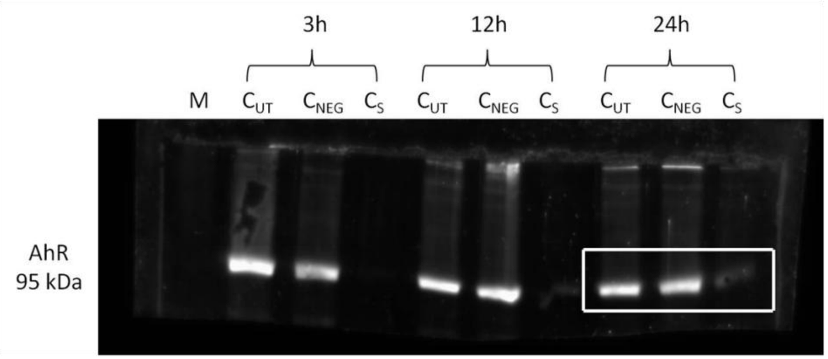

Full length Western blots corresponding to the panels shown in Figure 1C (24h).

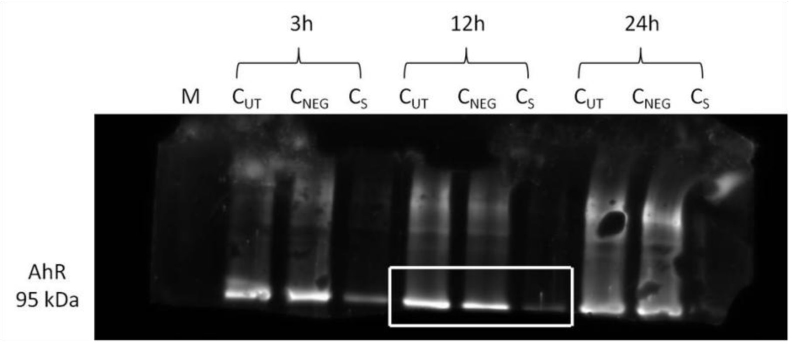

Full length Western blots corresponding to the panels shown in Figure 1C (12h).

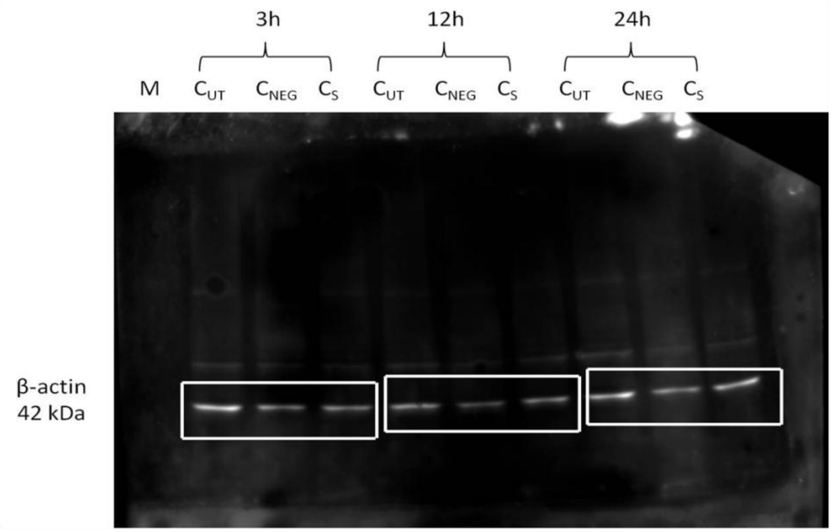

Full length Western blots corresponding to the panels shown in Figure 1C (3, 12 and 24h).

Supplementary Figure 1. The effects of knock-down of *AhR* gene expression on AhR protein abundance in porcine granulosa cells. The cells were transfected with three different siRNAs (24 h) and then cultured for an additional 3, 12 or 24 h. AhR protein abundance was determined by western blotting (n=4 biological replicates per one time point). Unedited blots for Figure 1C in the manuscript; representative immunoblots showed in Figure 1C were cropped to white-line boxes. C_{UT}: control untreated cells; C_{NEG}: negative control – cells incubated with siRNA duplex with an irrelevant sequence (nontargeted siRNA); C_S: AhR knock-down cells incubated with the mixture of the three different siRNAs targeting *AhR* (anti-AhR1 + anti-AhR 2 + anti-AhR 3).
